# Comparative analysis of human mitochondrial methylome show distinct patterns of epigenetic regulation in mitochondria

**DOI:** 10.1186/1755-8166-7-S1-P86

**Published:** 2014-01-21

**Authors:** Sourav Ghosh, Shantanu Sengupta, Vinod Scaria

**Affiliations:** 1 Genomics and Molecular Medicine, CSIR Institute of Genomics and Integrative Biology, Mall Road, Delhi, India; 2 GN Ramachandran Knowledge Center for Genome Informatics, CSIR Institute of Genomics and Integrative Biology, Mall Road, Delhi, India

## Background

Understanding the epigenetic regulation of genes and their functional implications in physiological and pathophysiological states has been one of the emerging areas of genomics. DNA methylation and histone modifications across the nuclear genome have been recently extensively analyzed in this perspective. However, the mitochondrial epigenome, though has been discussed widely has not been analyzed at genome-scale resolutions. The recent availability of genome-scale epigenome datasets allowed us to analyse profiles of methyl cytosine across mitochondrial genomes.

## Method

We analyzed methyl-cytosine profiles as evident from Methylated DNA immunoprecipitation across 39 tissues and cell lines that were available as part of the NIH RoadMap Epigenomics project. The mitochondrial reference genome used in the current study was derived from the UCSC human genome build hg19. We used custom scripts to retrieve reads mapping to the mitochondrial genome.

## Results

Our analysis suggests that the general profile of methylated cytosines across the samples show a distinct pattern. This pattern was generally conserved across the datasets considered, with exceptions of a few regions which showed variability in methylation amongst datasets analyzed. We show that certain regions of the mitochondria could be differentially methylated in datasets which show distinct temporal and functional characteristics, like Brain and Blood. One such region harbors the loci associated with mitochondrial encoded NADH dehydrogenase (MT-ND6), variations in which are associated with neurological disorders. To date this is the first and comprehensive analyses of genome-scale methylation data for Human mitochondria.

## Conclusion

This study describes the first comprehensive map of methyl-cytosines across the Human mitochondrial genome.

